# Review of Clinical Questions Submitted to Norwegian Drug Information Centres Concerning Administration and Dosage to Older Patients of Relevance to Patient-Centric Care

**DOI:** 10.3390/pharmaceutics13010105

**Published:** 2021-01-14

**Authors:** Jan Schjøtt, Lillan Mo Andreassen, Gro Helen Dale, Charlotte Lorentze Stokes

**Affiliations:** 1 Department of Medical Biochemistry and Pharmacology, Haukeland University Hospital, 5021 Bergen, Norway; lillan.mo.andreassen@helse-bergen.no (L.M.A.); gro.helen.dale@helse-bergen.no (G.H.D.); charlotte.lorentze.stokes@helse-bergen.no (C.L.S.); 2 Regional Medicines Information and Pharmacovigilance Centre (RELIS Vest), Haukeland University Hospital, 5021 Bergen, Norway; 3 Department of Clinical Science, Faculty of Medicine and Dentistry, University of Bergen, 5020 Bergen, Norway

**Keywords:** administration, dosage, drug, formulation, older, pharmacokinetics, patient-centric

## Abstract

Patient-centric care entails optimising healthcare provision to patients based on their perspective and opinion. It involves appropriate treatment at a reasonable cost and a focus on patient characteristics in the decision-making process to make it more personally useful. The optimisation of medicines in the older population is a challenge due to physiological changes, comorbidity, and polypharmacy. Furthermore, patient-centric care is difficult to achieve due to the high proportion of patients with dementia and frailty. Decision support concerning the appropriateness of indication, formulation, dose, administration, co-prescribing, and length of treatment to older patients is frequently in demand. In the current study, we aimed to review clinical questions concerning administration and dosage to older patients of relevance to patient-centric care. We analysed questions concerning medicines to patients 65 years or older in the database of the network of Norwegian drug information centres from 2010 to 2020. The analysis included the distribution of drugs, diseases, and recurring topics among the questions. Through a Boolean search that combined the indexed categories of “older” and “administration and dosage”, we retrieved 84 question-answer pairs. Questions about psychotropic and cardiovascular drugs in relation to therapy, adverse drug reactions, and pharmacokinetics dominated, and more than 60% of the questions came from physicians. Topics relevant to patient-centric pharmacotherapy were drug withdrawal (10 questions), drug formulation (8 questions), drug initiation (8 questions), and switching drugs (5 questions). One question concerned drug withdrawal and switching, and one question drug formulation and switching. Answers provided decision support regarding appropriate formulations of drugs to patients with dementia who chew capsules or tablets, the use of parenteral administration in patients who refuse to take oral formulations, and the pharmacokinetics of transdermal or rectal drug administration. The results highlight the importance of including pharmacological factors in the assessment of the acceptability and appropriateness of oral and parenteral medicine to older patients.

## 1. Introduction

Patient-centric care entails optimising healthcare provision to patients based on their personal perspective and opinion [1,2]. In a patient-centred approach, the health care providers have a responsibility to focus on optimising treatment according to the patient’s individual characteristics [3,4]. In patient-centric care, the source of information and healthcare planning is the patient themselves, while for patient-centred care the focus is on the role of the health care provider who seeks to optimise treatment through the use of tools such as technology and diagnostic measures. Both approaches could have beneficial consequences for patients’ quality of life, health, and health economics. An example of this is the costs related to patients’ nonadherence to prescribed therapies, which are estimated to be approximately US$100 billion per year [5]. A study from Canada of 311 family practice patients revealed that the costs of diagnostic tests decreased over four quartiles of patient-centred scores; the more patient-centred the visit, the less the cost of diagnostic testing in the two-month follow-up period [6]. The distinction between patient-centric and patient-centred is not clear cut when treating older patients due to several reasons. In Norwegian nursing homes, about 80% of patients have dementia, and communication is based on the observation of patients by healthcare providers and relatives [7,8]. Furthermore, it is estimated that 25–50% of people over 85 years old are frail and have a significantly increased risk of falls, disability, long-term care, and death [9]. Frail patients are exposed to periods of acute confusion (delirium) and reduced awareness. Older patients are associated with drugs and diseases that hamper awareness and communication [10].

The optimisation of medicines in the older population is a challenge due to physiological changes, comorbidity, and polypharmacy. Appropriate indication, formulation, dose, administration, co-prescribing, and length of treatment are frequent clinical issues. Patient-centric pharmaceutical drug product design, formulation, and optimisation for the older population is of current interest [11]. However, scientific progress in this field has to take into account the heterogeneous population and the complexity associated with medicine optimisation. Medication management with regard to older patients often becomes very complex due to the high number of drug products, dosage forms, and dosing frequencies. A cohort study of 855 community-dwelling patients aged 70 years or older with multimorbidity in Ireland found non-adherence in 31% of patients, but with adherence levels varying across treatment categories and chronic conditions [12]. Low adherence in patients with chronic medical conditions has economic [5] and clinical consequences. An observational study from the US found a link between partial or low statin adherence and all-cause mortality among patients aged 80 years or older [13]. A particular challenge is that individual pharmacodynamic and pharmacokinetic changes in older patients are not easily assessed. A Norwegian study of patients older than 65 years found significantly higher absolute serum concentrations for several antidepressants compared to the levels in younger patients. In this observational study, the concentration to dose ratios of antidepressants in patients older than 65 years showed a lack of sufficient dosage reductions to account for the increased systemic exposure [14].

Norwegian drug information centres provide decision support for drug-related questions from health care professionals in four health regions serving the total Norwegian population. In a previous study, we found frequent questions concerning psychopharmacology and cardiovascular pharmacology concerning older patients [15]. In the current study, we aimed to review clinical questions concerning administration and dosage to older patients of relevance to patient-centric care. The analysis included the distribution of drugs and diseases and recurring topics among the questions.

## 2. Materials and Methods

### 2.1. Data Material

Regional Medicines Information and Pharmacovigilance Centres (RELIS) is a Norwegian network of drug information centres providing decision support for health care professionals (e.g., physicians, pharmacists, nurses) in four health regions. The centres are associated with clinical pharmacology units in regional university hospitals, and pharmacists and physicians with expertise in searching and critically evaluating literature constitute the staff in the centres [16]. RELIS provide written answers with references and store indexed questions-answer pairs (Q/As) in a full-text, searchable database [17]. Selected Q/As are published online open access and are freely available to healthcare professionals. Answers considered as not having any general interest or questions that are so specific that there is a risk the patient can be identified are only available to RELIS staff. The Q/As are indexed with occupation (e.g., physician, pharmacist, nurse) and workplace (e.g., general practice, hospital) for each inquirer. From 2010, questions concerning older patients (≥65 years) were specifically indexed with the category “older” in the RELIS database. The database contains a search function for indexed drugs (e.g., generic name or Anatomical Therapeutic Chemical (ATC) code), 15 indexed categories (e.g., older, administration and dosage, interaction, adverse drug reaction), and text words (e.g., formulation, dementia). Thus, a Q/A can have several indexed drugs and categories. Simple, individual searches can be combined with Boolean operators (AND/OR/NOT) in the database.

### 2.2. Data Analysis

We performed a Boolean search combining the category “older” with the category “administration & dosage” in the period 1 January 2010–30 September 2020. Q/As from all types of healthcare occupations and workplaces were included, since patient-centric care involves communication with, and observation of, patients in general practice, pharmacies, nursing homes, and hospitals [2]. The result of the search was further classified by the authors, who included a geriatrician (C.S.), a pharmacist (L.M.A.), a junior (G.H.D.), and a senior (J.S.) consultant in clinical pharmacology. The authors registered indexed information about each inquirer in the Q/As and about the patient (if the question was not general concerning many patients or own practice) by examining the respective text. The drug(s) in the Q/As were classified according to the WHO Anatomical Therapeutic Chemical (ATC) system [18]. The WHO ATC system allocates drugs to different groups according to the organ or system on which they act and their therapeutic, pharmacological, and chemical properties. In the ATC system, drugs are classified in groups at five different levels, where the ATC 1st level has fourteen main anatomical or pharmacological groups and the ATC 5th level denotes the chemical or generic substance. Diseases associated with the Q/As were classified according to the WHO International Classification of Diseases (ICD-11) system mortality and morbidity statistics [19]. The authors also registered other indexed categories (e.g., adverse drug reaction, interaction) associated with the Q/As in the database, and suggested descriptive categories (e.g., drug initiation, drug withdrawal) to define recurring topics where appropriate. The Q/As were divided equally among the authors (J.S., L.M.A., G.H.D., C.S.) and classified into a common predefined data file, which was subsequently reviewed by J.S. Recurring topics with examples of relevance to patient-centric care were selected by J.S. after approval by all authors.

Descriptive statistical analyses were performed with SPSS version 26 (IBM Corp, Armonk, NY, USA). Percentages were not rounded up to 100 in either text or tables. Notice that the same question can include several drugs, indexed categories, descriptive categories, and diseases. Several drugs in a question can belong to the same main ATC group (ATC 1st level). ICD-11 can also include symptoms, and some questions concern the use of drugs in relation to procedures such as surgery or diagnostic measures.

## 3. Results

### 3.1. Question-Answers Pairs

The database contained 51,867 Q/As by the end of September 2020, and RELIS handled 3305 questions in 2019. In the study period, the size of the RELIS database increased from 19,785 to 51,867 Q/As. Two Q/As were excluded from the 86 Q/As retrieved by the Boolean search. In one Q/A, the patient was younger than 65 years, and in the other Q/A the category “older” was indexed when the appropriate category was “adverse drug reaction”. The final 84 Q/As were relatively evenly distributed (4–11 per year) over the study period, with the exception of a peak of 18 questions in 2015.

Fifty-four questions (64%) came from physicians and 25 (30%) from pharmacists. Three questions (4%) came from nurses, one (1%) from a dentist, and one (1%) from a patient relative. The four major workplaces were general practice (GP) and pharmacies, with 24 questions (29%) each; nursing homes with 18 questions (21%); and hospitals with 14 questions (17%). 

Sixty-three questions (75%) were patient-related, and 21 (25%) were general. Among the patient-related questions, 35 questions (54%) concerned women, 13 questions (21%) concerned men, and in 16 questions (25%) gender was unknown. Regarding age, 18 (29%) of the 63 patient-related questions concerned patients in their eighties, 9 questions (14%) concerned patients in their seventies, 8 questions (13%) concerned patients in their nineties, and 5 questions (8%) concerned patients in their late sixties (≥65 years). In 23 (37%) patient-related questions, age was unknown. Three of the general questions (14%) concerned patients older than 80 years, and one question (5%) concerned a patient older than 95 years. In 17 general questions (81%), age was not described. 

### 3.2. Drugs

The eighty-four questions concerned 129 brand drug names that involved 82 chemical or generic substances (ATC 5th level). The two most frequent generic substances were acetylsalicylic acid (6 questions) and metoprolol (5 questions). According to the WHO ATC system, the four most frequent ATC groups (ATC 1st level) were N (nervous system) with 53 counts, B (blood and blood-forming organs) with 24 counts, A (alimentary tract and metabolism) with 18 counts, and C (cardiovascular system) with 14 counts. Table 1 presents all 10 ATC groups identified in the material and the most common generic substances (ATC 5th level, appearing in ≥3 questions).

### 3.3. Diseases

The 84 questions concerned 104 diseases and three procedures (e.g., surgery or diagnostic measures). According to WHO ICD-11, the questions covered 13 codes, of which the majority concerned mental, behavioural, or neurodevelopmental disorders (24 questions), diseases of the circulatory system (20 questions), diseases of the digestive system (11 questions), and diseases of the genitourinary system (11 questions). See Table 1 for details.

### 3.4. Indexed and Descriptive Categories

The 84 Q/As were indexed 90 times across eight categories (Table 2), such as treatment, adverse drug reaction, and pharmacokinetics (i.e., some Q/As were indexed with more than one category). Twenty-two of the 84 Q/As (26%) had no additional indexed categories besides “older” and “administration & dosage”. Thirty-eight Q/As (45%) had one additional indexed category, 20 Q/As (24%) had two, and 4 Q/As (5%) had three. Three of the indexed categories (children, pregnancy, lactation) in the database were not relevant in this material, whilst the two remaining indexed categories (alternative medicine, other) did not occur in the 84 Q/As. The authors used four descriptive categories that were added 35 times to the 84 Q/As (i.e., some Q/As were classified with more than one descriptive category). Fifty-one of the 84 Q/As (61%) had no descriptive categories, 31 Q/As (37%) had one, and 2 Q/As (2%) had two descriptive categories. Table 2 presents the distribution of the indexed categories (besides “older” and “administration & dosage”) and descriptive categories (recurring topics) in more detail.

### 3.5. Examples

Table 3 shows examples of question-answer pairs (Q/As) relevant to patient-centric care.

## 4. Discussion

In this descriptive study, we aimed to review clinical questions concerning the administration and dosage of medicines to older patients of relevance to patient-centric care. The search in the RELIS database showed that questions about psychotropic and cardiovascular drugs in relation to therapy, adverse drug reactions, and pharmacokinetics dominated. More than 60% of the questions came from physicians. Our results are in accordance with a previous study on questions concerning older patients in the same database from the period 2010–2015 [15]. Drug-related questions are expected to be associated with frequently prescribed drugs, and group N in the ATC system came in third place (after groups C and A) in sales by million defined daily dose (DDD) in Norway in 2019 [20]. In our results, drugs from ATC groups B and A were frequently in question. These include, among others, drugs used for the prophylaxis of thrombosis (group B) and osteoporosis (group A), in addition to the drug treatment of obstipation (group A). Notably, psychopharmacology (group N) represents a particular challenge for physicians, and we believe that this is the reason for the frequent questions about psychotropic drugs submitted to Norwegian drug information centres in the last two decades [17,21,22]. When we compared the questions to RELIS in the period 1995–1999 with the period 2010–2014, the distribution of the major drug classes (described above) according to the ATC system did not change [22]. Furthermore, similar distributions have been reported by other European drug information centres [23,24]. Note that analgesic drugs such as paracetamol, tramadol, buprenorphine, and fentanyl, frequently found in the present results, also belong to ATC group N. These drugs are associated with the WHO ICD-11 category symptoms, signs, or clinical findings not elsewhere classified, and this category includes pain.

We do not know to what degree the distribution of drugs in the questions is influenced by factors such as the availability of authorised product information, professional handbooks, other drug information sources or the size of the portfolio of drug products. Although the pharmacokinetic information on the formulations in the summaries of product characteristics (SmPCs) is quite comprehensive, physicians still infrequently use this source [25]. Furthermore, RELIS frequently receive questions where discussions of statements in the Norwegian Drug Monograph (the short version of SmPCs) are in demand. A hypothesis is that the observed distribution reflects that drugs for the alimentary, cardiovascular, central nervous, and blood and blood forming organs are considered challenging to prescribe in a setting of old patients with a high prevalence of polypharmacy, and that neither clinical guidelines nor product monographs can provide sufficient decision support. Moreover, the population is associated with comorbidity that favours the use of prophylactic drugs mentioned above.

Adverse drug reactions, treatment, and pharmacokinetics are frequent reasons for questions to drug information centres [22,23,24]. The recurrent topics in the descriptive categories in our results further defined the clinical challenges. Drug withdrawal effects are in particular associated with psychotropic and cardiovascular drugs, and older patients are at risk due to reduced homeostatic capacity. Furthermore, drug formulation is increasingly important in the patient-centric care of older patients [11]. Due to the high risk of adverse drug reactions and interactions in this population, questions concerning drug initiation and drug switching is common.

Our examples reflect the problem with eating disturbances and drug intake among patients with dementia. Eating habits such as chewing or sucking without trying to eat is associated with mild to moderate dementia, while swallowing disturbance becomes critical in severe disease according to an observational study of 200 patients with Alzheimer’s disease [26]. Our examples with patients who chew capsules show the importance of assessing pharmacological factors in decision support concerning acceptability and appropriateness of oral medicine. Patients with cognitive impairment and dementia should be closely observed for signs of adverse drug reactions (ADRs) and/or ineffective drug treatment, since these patients are often unable to verbally communicate any treatment-related discomfort.

Dysphagia is common in the elderly [27] and is a known ADR to psychotropic drugs, in particular antipsychotics [28]. A study found that 1 in 11 primary care patients had frequent difficulties in swallowing tablets and capsules, while GPs grossly underestimated these problems [29]. Several strategies to ease the administration of solid oral dosage forms (SODFs), such as tablets or capsules, to older adults have been suggested [27]. The acceptability of SODFs is important for adherence among home-dwelling patients, and for drug safety (e.g., the risk of penetration, aspiration) among patients in nursing homes and long-term care. The examples of questions to RELIS included the highly prevalent prescribing of supplements with calcium and vitamin D. The various formulations of these supplements available today can provide solutions to patients with dysphagia.

The long-term use of prescribed and over-the-counter effervescent formulations is reported to be frequent among older people according to a study from the UK [30]. A possible reason is that these formulations increase patient acceptability. In a nested case-control study of a cohort of 1,292,337 patients with a mean follow-up time of 7.23 years, exposure to sodium-containing formulations of effervescent, dispersible, and soluble medicines was associated with significantly increased odds of adverse cardiovascular events compared with standard formulations of those same drugs [31]. Old people have impaired homeostasis and reduced renal function, which influence the control of blood electrolytes. With respect to this, our examples show that health care professionals should be aware of the constituents of effervescent formulations and toothpaste.

The perception that the absorption and bioavailability of transdermal and rectal formulations are limited in older patients could potentially reduce the use of alternative routes of administration. Transdermal formulations are particularly useful for the administration of analgesic drugs to patients with dysphagia, and the formulations are now available in several strengths to allow titration of dose. RELIS have received questions about cutting patches to allow titration, but we warn against this practice. A case reported in 2013 to the Institute for Safe Medication Practices (ISMP) described a physician who instructed staff from a hospice health care agency to cut a 50 mcg/h fentanyl transdermal system patch in half and apply it to a patient to deliver a 25 mcg/h dose [32]. A consequence of this practice is risk of overdose and serious harm to patients, in particular if the patches contain potent drugs such as fentanyl.

RELIS receive frequent questions concerning the switching of psychotropic drugs. In a commentary from 2016 we described several risk factors associated with this therapeutic intervention. Our experience is that physicians and other health care professionals are unaware of withdrawal reactions to old drugs, early-onset ADRs to new drugs, and that they lack knowledge about the role of active metabolites and interactions during switching [21]. The question concerning the transition from perphenazine to quetiapine reflects our general recommendation of “start low-go slow” when using psychotropic drugs among older patients. RELIS are associated with clinical pharmacology units that perform therapeutic drug monitoring (TDM), and in Norway it is possible to monitor a broad repertoire of psychotropic drugs [33]. As previously mentioned, observational studies of TDM results have found lack of sufficient dose adjustments of psychotropic drugs among older adults [14].

When interventions of treatment and care are carried out despite the resistance of the patient and/or against the patient’s will or knowledge, this is termed as coerced treatment and care. [34]. A study in home-dwelling persons with dementia in Norway found that physicians were usually responsible for the decisions, and nurses and family members were often involved in the process. However, family participation in coerced treatment and care raises ethical dilemmas [35]. One of our examples was a question from a hospital physician where the ethical dilemma of coerced medication was combined with the risk of exposing a patient to serious ADRs. The communication between the inquirer and RELIS focused on patient dignity when solving the problem.

Length of prophylactic treatment in older patients is also a relevant ethical and clinical question. In our study, this issue concerned osteoporosis and dementia, among other diseases. A problem is that there are no clear age limits, and many of the drugs are proposed to be effective in old age. Thus, we receive numerous questions from physicians who want to discuss problems that the recommendations and clinical guidelines cannot answer. The example with intermittent use of acetylsalicylic acid where the physician was advised to weigh cardiovascular risk and strength of indication against acceptability on behalf of the patient, show that RELIS are aware of the principles of patient-centric care. The individual assessment of a patient and including the patient perspective is of importance in our work.

The material in this study is naturalistic and descriptive, with a risk of biased interpretation. The great number of topics presented excludes a thorough discussion of each. Notice that the answers to the questions are related to the year they were provided. Furthermore, questions to RELIS are spontaneous, and do not necessarily represent drug problems perceived by the general population of health care professionals. However, in comparison to spontaneous ADR reports, a question to RELIS is in the context of clinical decision support, and as such a more deliberate action on behalf of a health care professional with a motivation to provide the best possible treatment of their patients.

The examples from the recurring topics focused on pharmacological factors associated with different formulations and administration of generic substances. However, the RELIS database is searchable for brand names, and health care professionals often submit questions about choice or differences between brands. We are aware of potential differences between brands as well as generic copies, but RELIS are independent of the pharmaceutical industry and we usually avoid favouring any specific trademarks in our answers. The examples also raise several ethical issues, and RELIS use a disclaimer on all written answers to state that they are based on the available literature and other resources, and health care professionals have sole responsibility for using the content of the answers in their treatment of patients. Our credibility among health care professionals relies on the fact that they can safely ask us all sorts of drug-related questions knowing that RELIS have no control function with regard to their professional practice. If we receive questions from patients or relatives, we usually motivate them to discuss the questions with their treating physician. Accordingly, RELIS do not interfere in the physician–patient relationship.

Although the problems presented are known in patient-centric care, a review of our database could be useful to find relevant topics for drug information activities for clinicians and patients. In particular, this is because the topics concern everyday, real-world problems not sufficiently described in recommendations and guidelines. Our selection of questions concerning the administration and dosage of medicines in older patients could be used to propose topics relevant to patient-centric medicine design. Furthermore, relevant stakeholders, including the pharmaceutical industry, should be aware of the specific drug information needs with regard to rational use in a patient population with specific challenges.

## 5. Conclusions

Our review of the database of the Norwegian drug information centres highlights the importance of assessing pharmacological factors in decision support concerning the acceptability and appropriateness of oral and parenteral medicine in older patients. Drugs and diseases of priority include those concerning the nervous system, blood and blood-forming organs, the digestive system, and the cardiovascular system. Through examples, we have described practical solutions to drug-related problems. The practical solutions involve several principles of importance for patient-centric care and involve scenarios that could motivate patient-centric medicine design. Given the current focus on new design of medicine, our present topics with examples could generate hypotheses to be tested in clinical settings in future studies.

## Figures and Tables

**Table 1:** Distribution of indexed and descriptive categories among the 84 questions.

**ATC-Groups ^a^**	***n*** ** = 129 (%)**
N (nervous system)	53 (41)
B (blood and blood forming organs)	24 (19)
A (alimentary tract and metabolism)	18 (14)
C (cardiovascular system)	14 (11)
R (respiratory system)	7 (5)
J (anti-infective for systemic use)	4 (3)
H (systemic hormonal preparations, excluding sex hormones and insulins)	3 (2)
M (musculo-skeletal system)	3 (2)
L (antineoplastic and immunomodulating agents)	2 (2)
S (sensory organs)	1 (1)
**Generic Substances ^b^**	***n*** ** = 129 (%)**
Acetylsalicylic acid	6 (5)
Metoprolol	5 (4)
Apixaban	4 (3)
Fentanyl	4 (3)
Macrogol	4 (3)
Mirtazapine	4 (3)
Paracetamol	4 (3)
Buprenorphine	3 (2)
Cefalozine	3 (2)
Diazepam	3 (2)
Escitalopram	3 (2)
Olanzapine	3 (2)
Rivaroxaban	3 (2)
Venlafaxine	3 (2)
**Diseases ^c^**	***n*** ** = 104 (%)**
Mental, behavioural or neurodevelopmental disorders	24 (23)
Diseases of the circulatory system	20 (19)
Diseases of the digestive system	11 (11)
Diseases of the genitourinary system	11 (11)
Symptoms, signs or clinical findings, not elsewhere classified	9 (9)
Endocrine, nutritional or metabolic diseases	8 (8)
Diseases of the musculoskeletal system or connective tissue	5 (5)
Diseases of the respiratory system	4 (4)
Diseases of the nervous system	4 (4)
Neoplasms	3 (3)
Endocrine, nutritional and metabolic diseases	2 (3)
Diseases of the immune system	2 (3)
Certain infectious and parasitic diseases	1 (1)

^a^ Main Anatomical Therapeutic Chemical (ATC) groups (ATC 1st level) according to the WHO ATC system; ^b^ generic drugs (ATC 5th level) asked about in ≥3 questions; ^c^ diseases according to the WHO International Classification of Diseases (ICD-11). A question can have several ATC groups, generic substances, and diseases.

**Table 2:** Distribution of the indexed and descriptive categories among the 84 questions.

**Indexed Categories ^a^**	***n*** ** = 90 (%)**
Treatment	27 (30)
Adverse drug reaction	27 (30)
Pharmacokinetics	18 (20)
Interaction	6 (7)
Product properties	6 (7)
Literature and identification	3 (3)
Toxicity	2 (2)
Mechanism of action	1 (1)
**Descriptive Categories ^b^**	***n*** ** = 35 (%)**
Drug withdrawal	11 (31)
Drug formulation	9 (26)
Drug initiation	8 (23)
Drug switching	7 (20)

^a^ Indexed categories in addition to older and administration and dosage in the Regional Medicines Information and Pharmacovigilance Centres (RELIS) database; ^b^ Descriptive categories added by the authors. A question can have several indexed and descriptive categories.

**Table 3:** Examples of question-answer pairs (Q/As) relevant to patient-centric care among the 84 questions.

Question	Answer
Are oral depot capsules of valproic acid effective in a woman in her eighties with dementia and affective disorder who chews the capsules (from a physician at a nursing home, 2010)?	The depot effect will be lost by chewing or crushing the tablets. Alternatives are valproic acid as a mixture. However, granulates from the capsules can be mixed in drink or food that do not need to be chewed.
What is the effect of oral depot capsules of galantamine on a female patient in her eighties with dementia and affective disorder who chews the capsules (from physician at a nursing home, 2010)?	The depot effect will be lost by chewing or crushing the tablets. Change in the amount of drug absorbed could give adverse drug reactions or lack of effect later in the dosing interval. Try an ordinary tablet formulation of galantamine with two tablets in the morning and two in the evening.
Are there any better alternatives to treatment with oral bisphosphonates and supplement of calcium/vitamin D in a woman with osteoporosis, constipation and dysphagia? (from a general practitioner, 2018).	Constipation is a common adverse drug reaction of oral bisphosphonates. The patients should be switched to injection treatment. Supplement of calcium and vitamin D could be switched from 18 mm tablets to 14 mm, or more frequent administration of smaller tablets or dissolving the tablets in water.
How is the transdermal (from a pharmacist 2012, and 2013) or rectal (from a pharmacist, 2012) absorption and bioavailability of drugs altered in older patients? Some physicians discuss the need of switching to oral formulations among the oldest.	Absorption and bioavailability are not significantly reduced. Dose adjustments should rather be based on assessment of individual systemic pharmacodynamic- and kinetic changes.
How to switch from perphenazine tablets, 8 mg morning and 4 mg evening, to quetipaine tablets in a female patient with auditory hallucinations (from a general practitioner, 2013)?	Both drugs have short half-lives that would imply the elimination of perphenazine and reach a steady state of quetiapine within 2–3 days. However, there is a ten-times difference in potency between the two drugs and the switch concerns an old patient. A gradual reduction in perphenazine and a gradual increase in the dose of quetiapine over 11 days was recommended.
Can effervescent tablets of paracetamol and acetylcystein be administered to a patient with hyperkalemia and renal failure (from a home care nurse, 2014)?	Effervescent tablets of paracetamol do not contain potassium. However, the use of effervescent tablets should be restricted in older patients due to their high sodium content.
Are there drug safety issues with the use of sodium fluoride toothpaste to patients in nursing homes who swallow it (from a dentist, 2015)?	Daily intake of sodium fluoride in this manner could reach up to 15 mg, well above the recommended daily dose.
Can we use an injection of olanzapine for a patient with dementia who refuses to take tablets when guidelines warn against the risk of stroke with second-generation antipsychotics (from a hospital physician, 2018)?	Injection treatment with olanzapine, which the patient responded well to, was continued to maintain the dignity of the person.
Are there any better alternatives to oral supplementation of calcium/vitamin D for patients older than 95 years with dysphagia after femoral neck fracture (from a general practitioner, 2019)?	There are no clear upper age limits with regard to prophylactic treatment. Vitamin D is available in droplets, and calcium is available as effervescent tablets or chewable tablets that can be mixed in yoghurt.
What is the antiplatelet effect in a patient who changed drug regime from acetylsalicylic acid 75 mg each day to every other day and experienced less stomach pain (from a pharmacist, 2020)?	It has been estimated that 20% of the platelets will have normal function with this regime. The physicians should weigh cardiovascular risk and strength of indication against acceptability on behalf of the patient.

## Data Availability

Data supporting reported results are available via contact to Regional Medicines Information and Pharmacovigilance Centres (RELIS), relis@helse-bergen.no.
